# A Future Perspective on Waste Management of Lithium-Ion Batteries for Electric Vehicles in Lao PDR: Current Status and Challenges

**DOI:** 10.3390/ijerph192316169

**Published:** 2022-12-02

**Authors:** Vongdala Noudeng, Nguyen Van Quan, Tran Dang Xuan

**Affiliations:** 1 Graduate School of Advanced Science and Engineering, Hiroshima University, 1-5-1 Kagamiyama, Higashi-Hiroshima 739-8529, Japan; 2 Ministry of Natural Resources and Environment, Dongnasok-Nong Beuk Road, P.O. Box 7864, Vientiane XHXM+C8M, Laos; 3 Center for the Planetary Health and Innovation Science (PHIS), The IDEC Institute, Hiroshima University, 1-5-1 Kagamiyama, Higashi-Hiroshima 739-8529, Japan

**Keywords:** lithium-ion batteries (LIBs), end-of-life (EoL), recycling, environmental impact, electric vehicles (EVs)

## Abstract

Lithium-ion batteries (LIBs) have become a hot topic worldwide because they are not only the best alternative for energy storage systems but also have the potential for developing electric vehicles (EVs) that support greenhouse gas (GHG) emissions reduction and pollution prevention in the transport sector. However, the recent increase in EVs has brought about a rise in demand for LIBs, resulting in a substantial number of used LIBs. The end-of-life (EoL) of batteries is related to issues including, for example, direct disposal of toxic pollutants into the air, water, and soil, which threatens organisms in nature and human health. Currently, there is various research on spent LIB recycling and disposal, but there are no international or united standards for LIB waste management. Most countries have used a single or combination methodology of practices; for instance, pyrometallurgy, hydrometallurgy, direct recycling, full or partial combined recycling, and lastly, landfilling for unnecessary waste. However, EoL LIB recycling is not always easy for developing countries due to multiple limitations, which have been problems and challenges from the beginning and may reach into the future. Laos is one such country that might face those challenges and issues in the future due to the increasing trend of EVs. Therefore, this paper intends to provide a future perspective on EoL LIB management from EVs in Laos PDR, and to point out the best approaches for management mechanisms and sustainability without affecting the environment and human health. Significantly, this review compares the current EV LIB management between Laos, neighboring countries, and some developed countries, thereby suggesting appropriate solutions for the future sustainability of spent LIB management in the nation. The Laos government and domestic stakeholders should focus urgently on specific policies and regulations by including the extended producer responsibility (EPR) scheme in enforcement.

## 1. Introduction

### 1.1. Lithium-Ion Battery Applications

Li-ion batteries (LIBs) have significant potential for energy storage use in appliances, heavy machines, and other facilities. They seem to be a substitute for lead-acid batteries and have begun to be used as a specific power supply for electric vehicles (EVs) [1]. Since LIBs have a longer lifespan than other types of batteries and possess higher densities of power compared with traditional battery technology, they are the first choice for consumers and manufacturers when considering applicability and efficiency. According to the research of the John Goodenough team, LIBs were released and first used in 1991 by the Sony company [2]. However, they were first proposed by M.S. Whittingham at Binghamton University in the 1970s. Currently, LIBs are widely used in many products in three major fields, including transportation, consumer electronics and devices, and grid energy and industry. The transport sector primarily uses LIBs in electric automobiles such as cars, auto-bicycles, buses, and forklifts [3]. Therefore, the demand for LIBs for power storage in EVs has continued to grow and is projected to increase about 17-fold, which will lead to the cost of battery storage going down in 2030 [4]. At present, there are several types of EVs, which are generally classified into four types, full electric vehicle or battery electric vehicles (BEV), plug-in hybrid electric vehicle (PHEV), hybrid electric vehicle (HEV), and fuel cell electric vehicle (FCEV).

### 1.2. EV Lithium-Ion Battery

At the beginning of the 21st century, the market for EVs is increasing year by year due to the imperative to meet global targets of reducing GHG emissions in order to combat global warming, improve air quality in urban areas, and respond to consumers [5,6]. LIBs have been developed as energy storage for the transport sector and renewable energy systems. Basically, a LIB consists of two cell electrodes, an anode and a cathode, and the main source of active Li-ions in a battery is the positive electrode (cathode). Based on the cathode materials, LIBs can be classified into different types, such as:

- Lithium Cobalt Oxide (LiCoO_2_)-LCO;
- Lithium Manganese Oxide (LiMn_2_O_4_)-LMO;
- Lithium Nickel Oxide (LiNiO_2_)-LNO;
- Lithium Nickel Manganese Cobalt Oxide (LiNiMnCoO_2_)-NMC;
- Lithium Nickel Cobalt Aluminum Oxide (LiNiCoAlO_2_)-NCA;
- Lithium-ion Phosphate (LiFePo_4_)-LFP;
- Lithium Titanate (Li_4_Ti_5_O_12_)-LTO (negative electrodes).

These types of batteries have different purposes when compared to multi-criteria, particularly energy storage capacity, price, reliability, safety, and lifespan [7,8,9]. Loganathan et al. analyzed multi-criteria of LCO, LMO, LNMCO, LFP, and LTO and found that the Lithium Titanate (Li_4_Ti_5_O_12_)-LTO battery was the best for EV applications [8]. The LTO is a state-of-the-art anode material because of its safety, abundant raw materials, great cycling stability, and stable charge and discharge voltage elevated level. However, in real status, EV models have mostly used NMC, NCA, LMO, and LFP batteries because of their stable crystal structure, abundant resources, and lower costs, as shown in Table 1 [3,10,11]. Nevertheless, various batteries retain limitations that should be improved or developed in the future to reduce production costs, increase capacities of application, and improve the EoL treatment technology. Currently, battery technology is continuing to develop in order to benefit from the use of EVs. Among prospective candidates, the solid-state battery is the most promising because of its high safety and high energy capacities [12].

## 2. Environmental Impact of EV LIBs 

### 2.1. Source and Pollution Pathways

EVs that use LIBs have advantages and benefits regarding environmental protection and pollution reduction, especially climate change impact mitigation, and help to limit GHG emissions. Despite the various advantages of LIBs, there are also drawbacks and risks to the environment and health from the improper treatment of EoL batteries, illegal disposal, and traffic accidents because LIBs include main components such as anodes, cathodes, and electrolytes. They also contain reactive salts, volatile organic, and other additives [13,14,15,16,17]. Therefore, if LIBs are decomposed or digested by various processes without proper treatment, hazardous and toxic chemicals will be released into the air, water, and soil, leading to harm to human health [18,19]. However, there are different chemical compositions of LIBs for different applications, depending on the manufacturer’s alternative. The typical chemical composition of LIBs mainly includes graphite (C), copper (Cu), aluminum (Al), cobalt (Co), nickel (Ni), manganese (Mn), iron (Fe), lithium (Li), organic carbonates, and plastic compositions. These chemical compositions are the significant sources that affect the environment and health when they are released into the atmosphere or ground via the life cycle [20,21]. Lithium cobalt oxide (LCO), lithium nickel cobalt manganese oxide (NCM), lithium iron phosphate (LFP), and lithium manganese oxide (LMO) batteries have critical components such as an anode, cathode, electrolyte, and separator. These components consist of electrochemical parameters and materials; each chemical and material can affect human health, as shown in Table 2 [22,23]. For instance, cobalt (Co) is a chemical element with characteristics as a solid, adaptable, and silvery gray color which is increasingly in demand in industrial sectors. Some spent LIBs contain large quantities of cobalt if released into the environment, leading to ecotoxicity and human toxicity in case of the highest accumulation or concentration [24]. However, LIBs mostly release hazardous chemicals to nature due to waste conditions under direct disposal and improper recycling process. 

#### 2.1.1. Soil Pollution

The main components of soil are 5–10% organic matter and 90–95% inorganic matter, which contains water and air. The composition of the soil varies depending on the kind of soil. Soil consists of divergent layers called soil horizons, including the top layer, subsoil, weathered parent rocks, and bedrock. The top layer is the most important for vegetation and crops because it is an organic component of soil. Major organic compounds include plentiful humus, lipids, saccharides, nitrogen-containing organics, and phosphorus-containing compounds [46,47]. The top layer of soil can be contaminated by toxic, hazardous wastes, especially heavy metals, from anthropogenic activities. The primary sources of soil pollution are industrial wastes, urban and domestic wastes, mining, agrochemicals, and atmospheric deposition [48,49]. Many cases of poor soil quality in urban and industrial areas have been observed by evaluating the toxicity of chemicals in soil. For instance, from 1994 to 2012, a significantly high concentration of heavy metals (Pb, As) was detected in the soil in the industrial district of Kaifeng City in China due to the rapid increase in vehicle and solid wastes [50]. Municipal solid waste disposed of in dump sites is a significant source of pollutants and the main contamination pathway of heavy metals/metalloids in soil, as in this study [51]. The highest concentration of metals such as Zn and Co in soil samples is mainly related to zinc-carbon and Co in spent LIBs. In recent years, EoL batteries from EVs have been increasing. If the waste batteries are not handled properly, they may lead to heavy metals contaminating the soil via the leaching of hazardous chemicals [52]. Thus, anthropogenic activities are a crucial indicator of increasing toxic, hazardous waste in soil from wastes of production processes and usage that, without proper management, impact human health via the food chain from the uptake of soil contaminants by plants or food crops [53].

#### 2.1.2. Water Pollution

Water pollution is the deterioration of water quality from substance contamination that makes it unsafe or adverse to humans, animals, and aquatic life. Water pollution is caused by anthropogenic and natural, anthropogenic processes, including industrial, agricultural, radioactive, and mining processes. The natural processes include pollution of water bodies by decomposed plants and animals and weather. Water pollution can be divided into three categories, groundwater pollution, surface water pollution, and seawater pollution, depending on the source and storage of water. The primary pollution sources are sewage and domestic waste, industrial effluents, agricultural discharge, soaps and detergents, toxic heavy metals, thermal pollution, and petroleum oil spills [47,54,55]. Municipal waste, domestic sewage, and industrial waste directly discharged without treatment significantly impact aquaculture and human health by contaminating the food chain with toxic heavy metals [56]. In the least developed and developing countries, municipal waste is mostly managed by collection and transfer for disposal in landfills [57,58]. Waste from municipalities increases the hostile environment in surrounding areas of the disposal site due to contamination from toxic metals (Zn, Cd, Mn, Ni, Mg, Pb) in electronic waste, including battery waste, into the groundwater [59,60,61]. In terms of EoL EV batteries, LIBs consist of heavy metals such as nickel, copper, and organic chemicals. If disposed of with municipal waste, they could lead to water pollution, which affects the environment and health [62]. 

#### 2.1.3. Air Pollution

Air is essential for humans, animals, and organisms living on earth. Pure, dry air contains 78.09% nitrogen and 20.94% oxygen by volume. Air pollution is caused by the contamination of organic, inorganic, and particulate matter that, in high concentrations, leads to harm to life, property, and heritage [63]. There are many sources of air pollution emissions, but they can be classified into the major sources: anthropogenic (industries, power plants, foundry units, etc.) and natural sources (volcanic, forest fires, storms, soil erosion, etc.) [64]. The major air pollutants that most affect human health include carbon monoxide (CO), particulate matter (PM), ozone (O_3_), sulfur dioxide (SO_2_), and nitrogen dioxide (NO_2_). In the Southeast Asia and Western Pacific regions, approximately 1.06 million people die yearly from indoor air pollution, and millions of people are affected by respiratory disease, which may be caused by exposure to ambient air pollution [65]. Industrial sectors are a significant source of air pollution released into the atmosphere, which causes toxic heavy metals in the air and soil in the areas surrounding the industries and leads to human health impacts, including cancer risk [66,67]. The transportation industry is the second largest source of GHG emissions, at 27% of all industries [68]. Increased GHG due to economic growth leads to high energy consumption. EVs are one of the best options for reducing air pollutants and mitigating the transportation sector’s GHG emissions [69]. Even though EVs are an essential technology for reducing GHG emissions and air pollution, EV batteries can pose a fire hazard if they are not disposed of properly or are involved in road traffic accidents, which might lead to emitting air pollution into the environment [16]. 

## 3. Current Treatment and Disposal of EV LIBs

**Figure 1:**
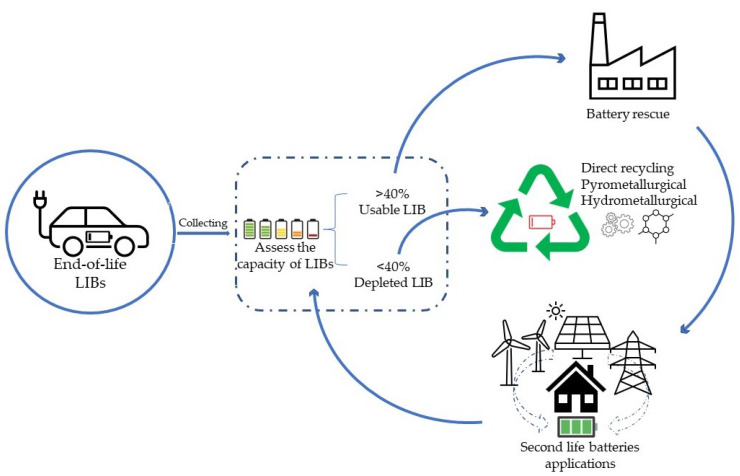
Main end-of-life LIB management processing by reuse and recycling.

The transportation sector has released approximately 14% of all direct and indirect GHG emissions, especially in Eastern, Southern, and Southeast Asia [70,71]. According to the Paris Declaration on Electro-Mobility and Climate Change and Call to Action, a target of at least 20% of vehicles globally being EVs, or more than 100 million cars, by 2030 has been set [72]. The global demand for EVs has increased. Additionally, the battery energy storage system is estimated to rise by 25% per annum, which leads to the supply risk of the materials or elements for manufacturing processes, especially elements for EV batteries production such as lithium, cobalt, graphite, manganese, and nickel [73,74,75]. Currently, LIBs are more prevalent in the global EV market due to having advantages such as high storage energy, long existence, etc. [76]. However, there are future challenges in dealing with the EVs’ spent batteries due to the need for recycling, treatment, and disposal technology to avoid pollution issues and establish economic sustainability [77]. EoL EV batteries can have a negative impact on the environment in various ways because they are comprised of toxic chemicals and hazardous materials. If improperly managed, they will release pollutants into the water, air, and soil. In this context, battery state-of-health (SOH) and recycling of spent LIBs (Figure 1) is a powerful method for reducing toxic waste entering the environment and reducing the need for virgin raw material resources [78,79]. Recycling technologies such as hydrometallurgical, pyrometallurgical, and direct recycling are most commonly used to recycle EoL LIBs [80,81,82]. Although there is varied research on LIB management, so far there are no international standards or united standards for waste disposal of LIBs in a unified, environmentally friendly way.

### 3.1. Recycling Methods

State of health (SoH) is a significant process to check the metric of battery operation features before considering them for reuse and forwarding them to the EoL battery recycling process. The SoH calculation monitors the remaining usable life of charge–discharge cycles, determining whether LIBs need to be replaced [83]. The machine learning method is the prevalent method of SoH estimation of LIBs in EVs and is accurate due to the diverse procedure for SoH estimation. The main process of machine learning methods includes data collection, feature extraction, model training, and SoH estimation [84,85]. Generally, when LIBs’ power capacity is less than 80%, they are not suitable for EV usage, and if the electric capacity is less than about 40%, they are no longer suitable for any commercial use. Thus, utilization of a battery could be effective when 40–80% of electric capacity remains [86,87]. 

The hydrometallurgical process of spent LIBs recycling is an efficient method for extracting valuable metals or materials from LIB waste through chemical methods, with the main processes of (1) dismantling and separation and (2) chemical processing and metals separation. The chemical process and metals separation use aqueous chemistry via acid leaching, chemical precipitation, solvent extraction, electrochemical separation, etc. This methodology can recover pure materials, uses low-temperature operation, and decreases GHG emissions better than the pyrometallurgical process. On the other hand, the separation of some elements, such as cobalt, nickel, manganese, iron, copper, and aluminum, is a high-cost and complex procedure using this method because these elements have similar properties [30,88]. However, there are many options and modern technologies for hydrometallurgical treatment, especially the leaching process, because leaching is a major step for metal extraction from EoL LIBs. In the hydrometallurgical operations for leaching procedures, there are multi-method options for application, such as high-pressure acid leaching, atmospheric pressure tank leaching, heap leaching, etc. These leaching procedures are the most suitable hydrometallurgical technologies for processing Ni-Co Lateritic Ores [89]. 

The pyrometallurgical process is a high-temperature method for dismantling spent LIBs to separate the alloy. Afterward, wet methods are used to leach the metals and carbon black. In this method, metals or metal compounds are furtherly separated to recover pure materials from a mixed metal alloy containing Co, Ni, Cu, Li, and slag containing Li, Si, Ca, Fe, and Al, which depend on the type and composition of the battery [90,91]. The pyrometallurgical process is a technology that is quite successful in terms of economic models for portable LIBs that have high cobalt content and are worthy or suitable for operation. However, EV batteries have lower cobalt content compared to portable batteries, which is less attractive for economic models. Nevertheless, this methodology has an uncomplicated process, and it is unnecessary to sort LIBs and NiMH batteries, which can be mixed in the recycled, mature technology. On the other side, there are disadvantages, such as the release of GHG emissions, high energy consumption, the need to increase the alloy recycling process, not recovering the utilization of the organic material, and being unsuitable for EVs’ battery recycling because of low Co concentration [88]. 

The direct recycling process recovers cathode material from LIBs by disassembling the battery and then recovering active materials for reuse or reconditioning for new batteries. In this process, the main procedures, such as the battery composition, are disassembled and materially separated via physical methods, and then the lithium source is replenished for compensation due to the degradation of the material during operation. Calcination or hydrothermal processes are needed for repairing the purified metals, and finally, the outcome of the process is regenerated active materials with multi-metals content [90,91,92]. The direct process has positives and negatives on the environmental and economic platforms; the uncomplicated procedure can use the materials from the final process directly and has low GHG and pollution emissions compared with pyrometallurgy and hydrometallurgy. However, there are some weak points in this process, such as sorting and pre-treatment needs for purified metal, the method has not been proven yet on an industrial scale, and it is an inflexible process [88]. Additionally, this technology is quite old and requires upgrading or improvement for the LIB recycling process.

In addition, in all of the methods mentioned above, there are still challenges and opportunities in the recycling process (Table 3); for example, using the pyrometallurgical method via incineration of the organic matter in LIBs with high heat provided mixed alloys of the valuable metal components in the results [93,94]. For this reason, a combination of pyrometallurgical and hydrometallurgical procedures is required in order to separate the mixed alloys to become pure metals. Currently, many recycling facility companies for LIBs worldwide still have limited detailed information; only some companies are releasing the information, such as Umicore (Belgium), Redex (Germany, Austria), and Duesenfeld (Germany) [95]. Even though there are recycling technologies for spent EV LIB treatment, these processes could affect the environment and health. Based on [96], a life cycle assessment of the hydrometallurgical method in a case study in China, highlighted qualitative results that the “endpoint impact categories”, global warming potential, and particulate matter formation potential have been contributors to damaging human health, and ecosystem quality. Nevertheless, the technologies presented were considered as net environmental benefits for reducing carbon emissions and human hazards and mitigating resource depletion, which leads to sustainable waste management [97].

### 3.2. Landfilling

Landfill is the term used to describe the primary disposal of municipal solid waste and hazardous waste management. The landfill methodology was the most economical and environmentally acceptable method at that time. The last decades have seen various changes in terms of the plan, design, operation, shutdown, post-closure control of landfills, monitoring, and economic utilization methodologies [99]. Currently, despite multi-changes methods for landfill operation, there is still the issue of environmental impacts from open-air burning, leachate, and gas due to old practices at sites that have not implemented the operating principles. Especially in developing countries, there are still various limitations to waste management because of a lack of cost operation, lack of access to innovative technologies, and lack of awareness and public promotions, which leads to improper management, such as unsegregated waste at the generation source. Mixed waste (hazardous waste and general waste) has been disposed of in open-loop landfills, which affects human well-being in the short-term and long term [100]. The low waste recovery rate and inadequate infrastructure of hazardous waste management have led to mixed MSW that consists of household hazardous waste, such as EoL portable LIBs and e-waste, still being discarded into landfills. It is a significant concern on environmental impact and toxicity to human health in landfill areas via released heavy metals to soil and leachate [70]. In terms of EV batteries, large LIBs have been recovered by recycling factories to reuse valuable metals, but in the final treatment process, there is still residue waste that is unusable such as bottom ash or invaluable waste; these are deposed into landfills. In recent decades, the EV market has grown rapidly, leading to inadequate facilities for EoL battery treatment in the future [101,102]. Thus, incineration and landfilling are still used for EV battery disposal after thorough treatment procedures; direct dumping of raw materials causes concern about increased environmental burdens and the possibility of food chain risks. 

## 4. Current EV LIB Management in the World

Scientific research has studied and developed the energy storage system (ESS) technology for transport sectors to mitigate air pollution and GHG emissions and reduce fossil fuel combustion on the earth. LIBs are the best alternative for the EV industry because of their high storage energy density, nominal voltage, lifespan, and environmental friendliness. In the last three decades, LIBs for EVs have been improved and developed to the state-of-art technology of the EV industry owner sectors by improving the higher storage of energy capacity, cost productivity of batteries, sustainability of use, etc. [3,103]. Furthermore, the metals and materials of EoL LIBs have been recovered from batteries’ bodies to reuse. Valuable metals recovery avoids the environmental and human health impact risks of direct disposal into the landfill without treatment and improper recycling processes. Spent LIB treatments and methods are different regionally and internationally, as there are no specific standards depending on industrial development, LIB supply chain, waste treatment strategies, recycling, etc. [93]. In 2010 there were 17,000 EVs, the number increased to 7.2 million EVs in 2019, and the trend is continuing to increase to 50 million by 2025 [104]. In addition, the International Energy Agency reported that the EV market increased to approximately 18 million vehicles in 2020 [105]. Thus, the world must invent or create modern technology for recovering and treatment of EoL EV batteries in order to save metals and materials and reduce pollution issues.

### 4.1. The United States

EV market growth in the United States (US) has increased annually and has become one-third of the market in the world; the number of EVs (new plug-in electric vehicles) sold was about 3.1 million in 2020, representing about 4.2% of light-duty vehicles. The number of Evs sold annually in the United States grew from a few thousand EVs in 2010 to 315,000 vehicles from 2018 to 2020. In addition, the government of the United States has declared a target of 50% electric vehicle sales share in 2030. To accomplish this, the government released policy and regulatory actions to push people to use Evs, including incentives such as reducing/exempting the EV tax and improving and providing facilities for EVs by increasing the infrastructure support and fast charging stations. In terms of regulations, states were issued Zero-Emission regulations [106,107]. The trend of EVs increasing led to the EV batteries supply chain being excessive. Global LIB trade in 2017–2019 showed that in the top five importer countries for over 51% of all imports worldwide, the U.S. imported 44% of the LIBs, while they are also exporters of lithium-ion batteries of 16% [108]. Due to battery expirations or end-of-life, LIBs must be managed properly to avoid environmental damage and recover valuable metals in the battery. The hydrometallurgy and pyrometallurgy systems for LIBs have been suggested and applied in some areas of the US [109,110]. Currently, the Resource Conservation and Recovery Act (RCRA) under the Environmental Protection Agency (EPA) and Hazardous Materials Regulation (HMR) under the Department of Transportation (DOT) have both been enforced in the US. The RCRA controls hazardous waste, consisting of generation, transportation, storage, treatment, and disposal, but the RCRA has not specifically identified LIBs and the HMR to control the safe and secure movement of hazardous materials in all modes of transportation. In addition, the DOT has also issued the Safety Advisory Notice for the Disposal and Recycling Lithium Batteries in Commercial Transportation in order to contribute to fully complying with US hazardous materials regulations [101,111].

### 4.2. European Union 

The European Union has set the goal to reduce carbon emissions via legislation and achieve climate neutrality by 2050. In this case, international and domestic transport using fossil fuels was considered to be about 30% of carbon emissions. In this respect, EVs are one of the alternative methods to reduce emissions from transport sectors in the EU [112]. The battery electric vehicles (BEVs) market has had an annually increasing trend, from approximately 700 units in 2010 to 550,000 units in 201,929 sold in European countries. In this context, plug-in hybrid electric vehicles (PHEVs) were about 1% and BEVs about 2% of total new vehicle registrations in 2019. This number accounts for electric vehicles’ market share of approximately 3.5% in the EU [113]. However, the total number of EVs registered in European countries reached 11,254,854 units for BEVs and 967,721 units for PHEVs, which showed that the amount of EVs has increased double [114]. Policy and regulations were developed by the European Strategy Energy Technology Plan (SET Plan) in order to accelerate the development of low-carbon technology with cooperation among EU countries and research institutions [115]. It also established legislative acts on Directive 2014/94/EU of the European Parliament and of the Council on 22 October 2014 on the development of alternative fuels infrastructure [116]. The EU has classified waste as hazardous or non-hazardous by commission notice guidance on the classification of waste (2018/C 124/01). It provides three categories of entries in the European List of Waste (LoW), absolute hazardous entries (AH), absolute non-hazardous entries (ANH), and mirror entries wastes. In this context, the mirror entries can be defined as two more related entries, such as one is mirror hazardous (MH) and the other is mirror non-hazardous (MNH). With regards to the battery waste stream, LIBs are under the section of other batteries and accumulators, which are classified as ANH waste [117]. The commission regulation (EU) No 493/2012 has noted that all waste batteries and accumulators should achieve the minimum recycling efficiencies set out in Directive 2006/66/EC. This ensures free-of-charge taking back of industrial and automotive batteries by the extended producer responsibility (EPR) scheme, which is part of Directive 2008/98/EC [118,119]. In 2020, the European Commission proposed a new regulation of batteries that would replace Directive 2006/66/EC in order to ensure that batteries placed in the EU market are sustainable and safe throughout their entire life cycle [120]. The EU reported that LIBs placed on the European market totaled 74,906 t in 2019, of which portable batteries of 49% and industrial batteries 51% were classified by the category set out in the Directive [121]. In terms of LIB recycling capacity, approximately 54,200 to 81,500 t/d in 2021 (in five European countries) were processed, mostly using the preferred three main processes, the combination of mechanical separation, pyrometallurgical, and hydrometallurgical methods [122].

### 4.3. Japan

The transportation sector in Japan contributes about 19% to entire CO_2_ emissions; in this context, clean energy vehicles (CEVs) such as battery electric vehicles (BEVs), plug-in hybrid electric vehicles (PHEVs), fuel cell electric vehicles (FCEVs), and hybrid electric vehicles (HEVs) are the main alternative for clean, renewable energy instead the internal combustion engine (ICE). In order to reduce emissions, the government planned to reduce CO_2_ emissions by 25% in 2030 [123]. However, the EV market in Japan is still quite growing slowly compared to China, the US, and the EU markets. Battery electric vehicles from Japan were sold in the worldwide market by approximately 5% in 2020. This is due to the focus on HEVs first and multi-factor conditions, for instance, a statement by automakers that the industry might “lose up to 200,000 jobs if all cars become fully electric or powered by fuel cells” [124]. The total number of BEVs and PHEVs sold in Japan was over 200,000 units in 2009 and continues to increase, while for FCEVs, the target number is approximately 40,000 units by 2020 and about 200,000 units by 2025 [125]. The Japanese government provided subsidies or incentives for the purchase of BEVs up to 800,000 yen/unit, PHEVs of 400,000 yen/unit, and FCEVs will be increased by more than 100,000 yen from the current 2.25 million yen/unit for the supplementary budget in 2020 [126]. For EoL battery management, there are no specified regulations on EV battery recycling but refer to the Act on the Promotion of Effective Utilization of Resources (2001), the Electrical Appliance and Material Safety Act, etc. [127]. Currently, there is no specific recycling regulation on EoL batteries from CEVs, but the government has promoted the extended producer responsibility (EPR) policy and encouraged consumers to send waste batteries to collection points [128]. Automakers such as Nisan cooperated with Sumitomo and 4R Energy set up (recycle, refabricate, reuse, resell) to recover electric car batteries [129], and in 2021 the DOWA ECO-SYSTEM Co., Ltd. set up new municipal waste and hazardous waste treatment facilities which can recycle LIBs [130]. Generally, LIB recycling technologies in Japan use multi-methods and are analogous to USA, EU, and China technologies which depend on the materials that need to be recovered from battery compositions. The Sumitomo-Sony method [131] has been used in the pre-processing, pyrometallurgy, and hydrometallurgy methods.

### 4.4. China

Presently, China is a top world market for EV sales and production, including battery manufacture [108]. According to the International Energy Agency (IEA), the EV stock globally will increase to 245 million units in 2030. In this context, EV batteries such as LIBs will also continue to increase in parallel with vehicle growth. The US and China are also leading countries in the EV batteries market [132]. The government has set up a plan to sell about 2 million EVs by 2020, 7 million by 2025, and 15 million units by 2030 [133]. Additionally, the government has formulated a strong policy of gently replacing internal combustion engine vehicles (ICEVs) with electric vehicles; the plan will be replaced and prohibit the sale of ICEVs as soon “as early as 2030”. The policy implementation showed that the number of sales has increased; for instance, for EV sales in China in January 2021, various models (ten models) were approximately 103,590 units [134]. There are many factors to encourage people to consider EVs, especially the subsidy policy in which the central government formulated consumer subsidies for consumer incentives [133]. The national EV policy incentive in China is a three-step strategy for electric vehicles industries with a formulated three-phase process, such as initiation in pilot cities (phase 1), expansion with significant financial subsidies (phase 2), and promotion to the whole nation but tightening subsidies regulation (phase 3) [135]. In terms of spent batteries or battery waste from EVs, the increase in EVs leads to a large amount of battery waste generation, with approximately 10,000 tons of EV LIBs in 2016. Based on predictions, EV battery waste will increase from 2012 to 2025 by about 700 to 464,000 tons [136]. China’s government adopted the extended producer responsibilities (EPR) system in order to promote EV manufacturing [137]. In reference to policy and regulations that are specifically related to LIB management, there are the main ones, such as the Guideline for Construction and Operation of New Energy Vehicle Power Battery Recycling Service Outlets and the Law of the People’s Republic of China on the Prevention and Control of Environment Pollution Caused by Solid Wastes (2020 Revision) [138,139], Notice on Printing and Distributing the Interim Measures for the Administration of Recycling and Utilization of New Energy Vehicle Power Batteries in order to implement the Environmental Protection Law and other laws and regulations [140]. Most of the EV batteries recycling methodology in China is also the same as other countries’ models, focusing on three recycling routes such as direct recycling, hydrometallurgical, and pyrometallurgical process [141]. However, the selection of recycling routes or technologies is dependant on batteries’ chemical compositions, cost-benefit, and other factors.

### 4.5. Thailand

In Thailand, EVs began to be promoted and manufacturing was developed in 2015. Presently, the EV market is growing and will show over 230,000 EVs in 2022, and the aim is to manufacture approximately 750,000 units in 2030 [142]. In the context, the International Energy Agency (IEA) released that EV sales in Thailand were 19,290 units from 2010 to 2019, and the government has a target of 1.2 million units on the road in 2036 [143]. In terms of EV battery manufacture, there is not still potential in the global supply chain for EV batteries because they are mostly a source of the EV battery assembly. However, based on predictions, the country will assemble about 430,000 units of EV batteries by 2023 [144]. In order to promote electric vehicle usage, the government has released an incentives policy, including lower excise tax and import duties; for instance, the excise tax on imported EVs is reduced to 2% from 8%, and manufacturers will also receive subsidies of about THB 70,000 to THB 150,000 for the EVs and THB 18,000 for electric motorcycles [145]. Regarding EoL battery recycling, there is no specific legislation for battery waste management, but there are legislations related to electronic waste management, such as the Environment and Conservation of the National Environmental Quality Act, B.E. 1992 [146] and the Public Health Act, B.E. 1992 [147]. However, with regard to [144], the responsibility for recycling and reuse of EV spent batteries in Thailand is unclear, especially who is responsible for the collection, testing, financing recycling, etc. The DOWA ECO-SYSTEM Co., Ltd., by Japan technology, has stated that in 2019 they will start recycling and treating battery waste from HV and EVs in Thailand [148].

### 4.6. Vietnam

EV consumer and market trends in Viet Nam are quite slow growing in the ASEAN region (except Cambodia, Laos, and Myanmar have not been compared). The number of EVs, including neighborhood electric vehicles (NEV), was 1086 units in 2016 and the target in 2020 is approximately 100,000 units [149]. However, the country is still new to EVs, with only 900 electric vehicle units on the road in 2020. The domestic automaker has released more than 50,000 orders between 2021 and 2022; however, the number of orders does not reflect real sales to date [150]. The electric mobility market in the country mostly still prefers e-bikes and e-motorcycles, which are domestic products. In 2018, almost 500,000 electric two-wheelers (E2Ws) were sold in the nation, but in contrast to the HEVs, PHEVs, and BEVs, there is a small number, approximately 50 units, imported from abroad [151]. The slow growth of EVs in Viet Nam due to many factors and conditions, especially since the government does not concentrate on this matter. Currently, there is no policy to encourage EV development and incentives for consumers, but private companies are still developing automobiles and motorcycles to become the leading manufacturer in Southeast Asia [152]. Regarding the management of spent EV batteries, there is no specific legislation on EoL battery management in Viet Nam, the e-bikes and e-motorbikes use lead batteries, and the electric vehicle’s batteries are LIBs. Both batteries were defined as hazardous waste (HW) according to Vietnamese legislation, such as Circular no. 36/2015/TT-BTNMT [151]. However, the real management and implementation of legislation in Vietnam are vulnerable for many reasons, for instance, the lack of specific regulations, facilities, and subsidies from the government to support the collection, recycling, and disposal activities [153]. Currently, there are no facilities for EoL EV battery recycling and recovery in Vietnam due to the smaller number of EV usage in the country, but in the next 10 years, it needs to prepare to deal with end-of-life EV battery management [154].

## 5. Current Management of EV LIBs in Lao PDR

### 5.1. EV Market and LIB Demand

Regarding electromobility, Laos’ policy toward EVs still lags behind other countries in the ASEAN region (except Cambodia and Myanmar, these countries were not compared) [149] even though the government started a program of promotion and awareness on EV usage in order to reduce GHG emissions and fossil fuel consumption in big cities [155]. The kind of electromobility vehicles used in the country mostly consists of e-bicycles, e-motorcycles, e-car, e-minibuses, and e-trikes. However, Laos is a small market for EVs because of its small and mostly low-income population [156]. Currently, there are ten companies approved by the government for the sale and import of EVs as well as charging station services. In the whole country, there were more than 300 units of electric vehicles in 2022 (not including e-bicycles and e-motorcycles); these are mostly imported from China. Based on the import and sale mechanism, the EV market will increase in 2022 by about 30–50% compared with 2021 [157]. There are two types of batteries used in electromobility vehicles in the country, lead-acid batteries and LIBs. Both types of batteries have been imported and promoted in the country depending on the kind of EV, but so far, light-duty electric vehicles are still a small market in Laos. Therefore, most EVs are e-bicycles and e-motorcycles which use lead-acid batteries due to their low cost compared to LIBs. There still is no comprehensive management system for EoL EV batteries, especially regarding regulations, recycling, and disposal [156].

### 5.2. Governmental Plan and Project

The Lao Government has pledged to reduce GHG emissions by 60% unconditionally from business as usual by 2030 (the nationally determined contributions to the Paris Agreement) [158]. In the context of GHG emissions, the transport sector is one of the main contributors to CO_2_ emissions from gasoline and diesel consumption in the country. The transport sector has grown every year, which leads to increased CO_2_ emissions. According to an estimate from the JICA study team, emissions grew from approximately 2,179,297 t CO_2_/year in 2011 to 3,832,668 t CO_2_/year in 2020 and will be 6,286,348 t CO_2_/year in 2030 [155]. In Laos, the CO_2_ emissions by sector can be divided into transport (36.9%), non-combustion (23.6%), building (18.4%), other industrial combustion (15.7%), and power industry (5.4%), respectively [159]. To reach the apparent goals of GHG emissions and air pollution reductions from transport sectors related to energy consumption, the government has formulated EV usage in the transport sector via the strategy of relevant sectors. The strategy has outlined the initiative scenario; EV usage in four big cities will cover about 30%, which equates to approximately 1.2 million electric vehicle units by 2030 [160]. In recent years, the government has released a new policy to promote electric vehicle usage to increase the number of EVs on the road to 1% by 2025 and over 30% by 2030. Apart from that, the country has promoted and allowed domestic and foreign entrepreneurs to develop EVs in Laos [155]. Even though the initial action of electric vehicle promotion is quite late, the government has had a short-term pilot project for the promotion of electric vehicle use in place since 2014 [161]. The project was implemented in two pilot cities, and the project model can continue to expand to other cities throughout the country. 

### 5.3. Challenges for EV Adoption in Laos

As noted, the government has reduced GHG emissions, air pollution, and energy consumption from the import of petroleum by promoting EV usage in the country. Nevertheless, in the last decade, a baseline data collection survey was conducted on the feasibility of EV usage in Vientiane and Luang Prabang cities, which was part of a short-term pilot project that was implemented [155,162]. In the beginning, it seemed likely that the project would go smoothly because its ambitions were supported by external expertise and facilities. After a few years, the project has had problems in real implementation because of many obstructive conditions to operation, especially (1) road conditions can damage the frame and battery of EVs and (2) in the rainy season, there is regular flooding in urban roads, these might lead to electric short-circuits of the electric vehicles. In addition, there will be challenges in the future with EoL EV battery management due to the lack of treatment facilities (EoL batteries material recovery), policies, and regulations [163]. Even though road infrastructure improvement is a significant need for EV convenience, it cannot be implemented in the short term due to the high cost of investment. Although there is an inadequate budget for road infrastructure investment, most infrastructure projects are funded by foreign sources (loans), which might reflect an accumulation of external debts [164]. Furthermore, EV penetration increases might lead to high electric demand, which needs financial support for power investment. Meanwhile, CO_2_ emissions will decrease due to reduced petroleum consumption, but this will have negative impacts on the revenue of oil companies [165]. As mentioned above, not only Laos has faced these challenges, but developing countries also [166]. 

### 5.4. Waste Management and Current Disposal Legislations

The Law on Environmental Protection No. 29/NA/2012 of Laos categorized waste into two types: general waste and toxic and hazardous wastes (including battery waste) [167]. The law defined principles for general waste management, including segregation for different purposes such as reuse, recycling, reprocessing as new products, treating as a technical process, and disposal based on regulations. Toxic and hazardous waste should be treated with techniques and disposed of within identified areas according to regulations. In practice, municipal waste management (MWM) still has limitations and issues, such as low collection rates and improper disposal, but it seems that they can be resolved and will be better in the near future due to cooperation and support for the project’s implementation from international + MWM is one priority task of national agenda. In contrast, with toxic and hazardous waste, there are still no adequate handling systems and a lack of waste data recording; even if some industrial zone areas progress to deal with hazardous waste, there are challenges with final disposal [168]. Hazardous waste management (HWM) in industrial factories is referred to as Decision No. 0555/IC.DOIH/2012 and divides industrial waste into two types: hazardous waste (HW) and non-hazardous waste (NHW), which determines the management of waste in industrial factories [169]. According to Decision No. 1687/MONRE/2021, the import of toxic, hazardous waste and waste such as chemically contaminated waste, batteries waste, e-waste, and radioactive waste, including waste in the list of the Basel Convention, is prohibited [170]. However, household hazardous waste (HHW) consisting of battery waste (including LIBs) from devices/electronic equipment has not been segregated and recycled but combined with municipal waste and disposed of in landfills [61]. EoL LIBs from EVs is still a new matter at present, but it is imperative to develop management mechanisms in order to cope with problems and challenges in the future. Until now, there is no specific regulation on battery waste management, which is a significant tool for EoL battery management from municipalities, industries, the energy sector, and spent batteries of EVs.

## 6. Challenges, Discussion, and the Way Forward

EVs still have many challenges and problems for real implementation in developing countries because of small-scale market structures, infrastructure networks, economies, and regulatory frameworks [171,172]. Laos is one such country. There are still many limitations to EV application in the country in order to encourage GHG emissions reduction, petroleum import abatement from aboard, and pollution control. In terms of EoL battery management, currently, the nation lacks potential technology for the reuse, recycling, and disposal of EoL lithium-ion batteries. There are many types of LIBs (their chemical compositions are shown in Table 4), which can lead to emitting pollutants into the environment and affecting health. However, there are still many unknowns and fragmentary data. The lack of current specific research and arguments on advantages and disadvantages results in difficulty in explaining how to reduce any negative impact of the spent batteries. Thus, it is necessary for logical thinking and identification of major viewpoints on LIBs and the environment and health risks of EoL batteries via some main questions in order to help to manage in the future:

1. What are spent LIB quantities in the present and future?
2. How to monitor the EoL LIBs’ flow chart in the country?
3. What, how, and where are elements or hazardous chemicals released from EoL LIBs?
4. How, where, and who handles LIB waste?
5. What is the mechanism of public and private participants for dealing with EV batteries?

All these main questions on the environmental and health impacts of LIB waste are challenges and could become crucial problems in the future if there are no countermeasures against it in advance. However, only these five questions can not respond to and cope with issues in the future; more research needs to be conducted. Therefore, it is necessary to clarify and answer the questions by referring to the current situation of Laos and abroad via literature review in order to modify and apply in the future. Although some of these questions have already been answered, there is still a need for clearer pointing; for instance, EoL LIB quantities at present are zero because the market for Evs is new in Laos. Thus, approximately 300 units of EVs/batteries are still in use and have not expired yet [157], with approximately 1.2 million in 2030 [160]. Nevertheless, the data have only shown electric vehicle cars, but there are no data on e-bicycles and e-motorcycles; this point is a significant gap that should be considered for EoL battery management in the country. 

The increase in EVs, in parallel with the growing demand for LIBs, might lead to an increased number of EoL batteries. Therefore, empowering governments to establish and develop policies, regulations, and standards for managing the environmental pollution issues from spent batteries in the future is significant. In addition, lithium-ion battery waste flows at present and in the future from EVs by using the material flow analysis (MFA) is needed to estimate the volume and stream of LIBs waste in Laos and to develop the plan for EV battery management, such as the reuse of battery cells and packs, infrastructure capability of recycling, and safe disposal routes planning [173,174,175]. It is necessary to know about battery composition and negative impact routes, as in Table 4, as the recycling and disposal process of spent LIBs may pose challenges for toxic chemical emissions to air, soil, and water leading to risks to environmental and health impacts. Meanwhile, the recycling industry still faces many practical obstacles currently, such as the technical limitations of different recycling processes, but generally, there are three main processes, which are shown in Figure 1. However, recycling spent LIBs has been implemented in developed countries and some developing countries that are electric vehicle automakers, such as China. In the case of Laos, there are many limitations to dealing with LIB waste management, so the extended producer responsibility (EPR) system is a significant and appropriate method to be applied in the country. As known, the EPR has a potential role in encouraging the safe and sustainable management of EV EoL batteries and also supports achieving a circular economy approach [75,176,177]. Thus, the government should consider developing specific policies, regulations, and standards on electric vehicle battery management to prevent improper battery waste management and mitigate the responsibility of the public. With regards to the mechanism of the public and private participants in spent EV battery management, both sectors should have closely cooperated after the government released the EPR policy or regulation, the private (seller or manufacturers) must ensure that they bear financial responsibility for the collection, recycling, and disposal of EoL batteries. Furthermore, advanced technology promotion is also significant for spent EV battery management, such as artificial intelligence (AI), the Internet of Things (IoT), and machine learning (ML) in order to support economically and environmentally sustainable (EES) recycling, state-of-health (SOH) battery monitoring, information sharing, traceability of LIBs, etc. [178,179]. 

## 7. Conclusions

The end-of-life (EoL) battery management of EVs is significant to avoid environmental and health impacts and to reduce the use of virgin raw material resources. Lithium-ion batteries (LIBs) contain multi-chemical compositions in their bodies, and direct disposal and/or improper recycling processes might release pollution into the air, water, and soil, leading to toxicity to the environment and health. Thus, the reuse, recycling, and disposal methodologies have been mostly applied for dealing with battery waste. The recycling methodology of EoL LIBs has been extensively researched in order to recover valuable metals and materials. However, the research still has some disadvantages that must be resolved in further development. The key mechanisms to manage the EoL batteries of EVs worldwide are (1) develop specific policies, regulations, and standards and (2) develop technologies for reuse, recycling, and disposal. Currently, there are different routes for battery waste recycling from EVs, depending on the metal and material compositions, but mostly there are three major processes: direct recycling, pyrometallurgical, and hydrometallurgical processes. 

Waste management in Laos has quite a low capacity for reuse, recycling, and disposal; LIB waste management, especially, has not been considered for systematic management so far. However, EoL batteries are increasing every year due to the EV market increasing, which means that EoL EV batteries must be managed in the future (at least in the next decade). However, the technology mechanisms of developed countries/automakers countries can not be applied or implemented directly due to the excessive cost of establishment and operations, which is not worthy of investment for low to middle-income countries. Thus, one major aspect that should be considered as an adaptation to apply properly in the future is developing specific policies and regulations for EVs via formulating the EPR scheme into policies and regulations. 

## Figures and Tables

**Table 1:** A brief summary of various Li-ion battery cell chemistries for EVs, adopted from [3,11].

EV Models	Battery Supplier	Cell Chemistry	
		Positive Electrode	Negative Electrode
Audi e-tron GT/Audi Q4 e-tron-SUV/BMW i3/Chevrolet Bolt/Hyundai KONA Electric/Hyundai ioniq 5-LR AWD/Kia Niro/Kia Soul EV/Kia EV6-LRAWD/Nissan Leaf Plus/Renault Zoe e-tech electrique/Smart fortwo Electric/Volvo XC40/VW eGolf/Volkswagen ID.3	LG Chem/Samsung SDI/SK Innovation/AESC/Sanyo/Li-Tec	NMC	C
Tesla Model S/Toyota rav4 EV	Panasonic	NCA	C
Tesla Model 3/Tesla X	Panasonic	NCA	SiO-C or Si-C
BYD Tang Electric/Chevrolet Spark EV	BYD/LG Chem/A123	LFP	C
Fiat 500e/Ford Focus Electric/Renault Zoe	Samsung SDI/Li Energy Japan/LG Chem	NMC-LMO	C
Ford Focus Electric/Mitsubishi iMiEV	LG Chem/Li Energy Japan	LMO-NMC	C
Honda fit EV	Toshiba	NMC	LTO
Nissan Leaf	AESC	LMO-NCA	C

**Table 2:** Key composition of LCO, NCM, LFP, and LMO batteries and health impact.

Main Component	Electrochemical Parameters and Material	Human Health Impact
Anode current collector,	Copper	The highest concentration of Cu led to cardiovascular, immunity, and nervous system risks [25,26].
Anode,		
Separator/Binders,	Graphite	High concentrations of graphite flakes affect mechanisms of the respiratory systems, and a minor risk of physical-mechanical damage to unprotected skin and eyes also exists, with human carcinogenic toxicity [27,28].
Cathode,		
Cathode current collector,	Plastic	Negative impact on body function and increased risk of disorders and diseases [29,30].
Electrolyte		
	Cobalt	Inflammatory lung, allergic skin reaction, etc. [31,32].
	Aluminum	Al toxicity causes harm to the brain system, bone marrow, and osteomalacia [33,34].
	Iron/Streel	Particulate matter of Fe leads to risks of cardiopulmonary diseases and stroke and increased vulnerability to inflammation-associated pathologies such as respiratory diseases and lung cancers [35,36].
	Lithium	High concentrations of Li cause toxicity to human cardiomyocytes, and itcan affect hematopoietic stem cell differentiation and glycogen synthesis during fetal development, etc. [37,38].
	Manganese	Mn nanoparticles can access the brain, causing damage to neurological syndromes, such as Parkinson’s disease [39,40].
	Nickel	Ni chronic exposure in the body has adverse negative health effects in humans, such as lung fibrosis, renal disease, cardiovascular disease, and respiratory tract cancer [41,42].
	Electrolyte	Electrolytes of LIBs usually include both organic solvents and inorganic solutions, can cause corrosive skin burns, eye damage, and produce hazardous gas, leading to respiratory systems [43,44,45].

**Table 3:** Challenges and opportunities of hydrometallurgical, pyrometallurgical, and direct recycling processes [94,98].

Methodologies	Advantages	Disadvantages
Hydrometallurgical	High recovery rate and high purity of valuable metals (including Li) Lower energy consumption Products could be used for cathode production Commercially available No air emissions	Presorting and pre-treatment required Embedded energy inside of material structure loss Lengthy and complex process High operating cost Pollutions (GHG, wastewater, etc.) caused by extensive usage of chemicals
Pyrometallurgical	Flexible input materials No presorting Short process flow Commercially available Could replace mining and metallurgy to a certain extent	Li and Al remaining in slag Embedded energy inside of material structure loss Extra cost for treating gas pollution Not targeted recycling and need metal separation High energy inputs Capital intensive
Direct recycling	Low cost, low consumption Less pollution More profitable Embedded energy inside of material structure maintained Product can be used directly as cathode materials	Presorting required with more separation processes More requirements for input materials (sin-gle materials recovery, degradation condi-tions, etc.) Regeneration processes yet to be developed Still at lab scale

**Table 4:** Overall LIB compositions and pollutant emissions [70].

Battery Component	Sources of Pollutant	Specific Pollution	Route	Affected Environment	Hazard
Pack casing	Steel	Fe, Al, Ni, Cr, other	Leaching	Land Natural waters	In excess toxic to wildlife Accumulation in plants and crops
Module casing	Steel Aluminum	Fe, Ni, Cr, other Al	Leaching	Land Natural waters	In excess toxic to wildlife Accumulation in plants and crops
Cell packing	Aluminum foil Polymers Ni-Coated steel	Al, Ni PET, PP	Leaching Fire	Natural waters Land Air	In excess toxic to wildlife Accumulation in plants and crops
Cathode	Metal Metal oxides	Al LMO—Li/Mn/O LFP—Li/Fe/P/O NMC—Li/Ni/Mn/Co/O LCO—Li/Co/O NCA—Li/Ni/Co/Al/O	Leaching Dust	Land Natural waters Air	Toxic to the various organism Toxic to humans if breathed In excess toxic to wildlife Accumulation in plants and crops
Anode	Copper Graphite	Cu C (nanomaterial) LTO—Li/Ti/O	Leaching	Land Natural waters	In excess toxic to wildlife Accumulation in plants and crops Toxic to humans if breathed Microplastics accumulation
Separator	Polymers	Polyethylene (PE) Polypropylene (PP)	Leaching Fire Dust	Land Natural waters Air	Microplastics accumulation
Binder	PDVF	HF	Fire	Air	Toxic to humans if breathed Toxic to humans if in contact
Electrolyte	Ethylene carbonate Propylene carbonate Dimethyl carbonate Diethyl carbonate Salts: LiPF_6_ Additives	HF	Fire Vapors/gases Leaching	Air Land Natural waters	Toxic to humans if breathed Toxic to humans if in contact Toxic to wildlife Accumulation in soils
		SOx			
		HCN			
		H_2_			
		CO			
		CO_2_			
		NO_X_			
		COS			
		HCl			
		Degradation products of electrolyte			
		C_2_H_4_; CH_3_COCHO etc.			
		Ionic liquids			
		Unknown additives/degradation products			
